# Camystat: a high-throughput open-source software tool analysis of contractile cardiomyocytes recordings

**DOI:** 10.1093/cvr/cvag103

**Published:** 2026-05-07

**Authors:** Marcin A Rojek, Piotr Lewandowski, Artur Morys-Magiera, Filip Pietryga, Aleksandra Kowalik, Sławomir Grzegorczyn, Romuald Wojnicz

**Affiliations:** Department of Histology and Cell Pathology, Faculty of Medical Sciences in Zabrze, Medical University of Silesia, ul. Jordanna 19, Katowice 40-055, Poland; Department of Biophysics, Faculty of Medical Sciences in Zabrze, Medical University of Silesia, ul. Jordanna 19, Katowice 40-055, Poland; Department of Histology and Cell Pathology, Faculty of Medical Sciences in Zabrze, Medical University of Silesia, ul. Jordanna 19, Katowice 40-055, Poland; Faculty of Electrical Engineering, Automatics, Computer Science and Biomedical Engineering Department of Automatic Control and Robotics, AGH University of Krakow, al. A. Mickiewicza 30, Krakow 30-059, Poland; Independent Researcher, Katowice, Poland; Silesian Park of Medical Technology Kardio-Med Silesia, M.Curie-Skłodowskiej 10c, Zabrze 41-800, Poland; Department of Biophysics, Faculty of Medical Sciences in Zabrze, Medical University of Silesia, ul. Jordanna 19, Katowice 40-055, Poland; Department of Histology and Cell Pathology, Faculty of Medical Sciences in Zabrze, Medical University of Silesia, ul. Jordanna 19, Katowice 40-055, Poland; Silesian Nanomicroscopy Center, Silesia LabMed—Research and Implementation Center, Medical University of Silesia, Zabrze, ul. Jordanna 19, Katowice 40-055, Poland

**Keywords:** Contractility analysis, hiPSC-derived cardiomyocytes, Binary differential analysis

**Time of primary review: 111 days**

*In vitro* analysis of contractile cardiomyocytes is one of the key approaches used for assessing drug-induced cardiotoxicity and modelling physiological and pathophysiological processes in cardiac tissue. Cardiomyocytes cultures are typically derived from two major sources: primary isolation (e.g. from neonatal rodent hearts) or differentiation of pluripotent stem cells, including human induced pluripotent stem cells.^1,2^ Regardless of their origin, the fundamental method for assessing cellular activity remains microscopic observation of contractility. In this context, the evaluation of contraction and relaxation frequency, intervals, and amplitude within groups of cells remains essential. Manual measurement of these parameters remains challenging, primarily due to the high contraction frequency and the inherent limitations of microscopic observation. To improve the objectivity of such analyses, numerous software tools have been developed for the assessment of cardiomyocyte recordings, available either as extensions to existing platforms or as standalone applications, with both open- and closed-source implementations.^3,4,5^ These approaches differ considerably in terms of the availability of a graphical user interface (GUI), the analytical algorithms employed, and the scope of output reporting, ranging from simple time-dependent activity measurements or contraction counts to detailed information on contraction and relaxation timing. In this context, there has emerged a need to develop software that could serve as a standardized tool, enhancing both the usability and reproducibility of studies involving contractile cardiomyocytes. Based on a review of existing solutions, we defined a set of criteria that software must meet in order to serve a standardizing role in cardiomyocyte research, and we developed Camystat as an open-source software tool designed for the automated analysis of cardiomyocyte recordings.

We developed Camystat as an easily accessible, ready-to-use software package distributed with executable installers, enabling installation and operation by users without prior programming experience. The software was implemented in C++ and Python, and the complete project documentation is available under the GNU General Public License v3.0 as open-source material on GitHub and the dedicated website camystat.myocore.io. The website also provides a comprehensive user manual, as well as installation packages for multiple platforms, including Windows, macOS, and Linux. C++ was used due to its low-level computational optimization capabilities, and Python was used for its Plotly library, which enables saving interactive visualizations. Our software features a GUI, an interactive plotting module capable of automatically detecting contractile and relaxation activity, a spatial analysis module that generates heatmaps of culture dynamics, and a data output module providing both raw activity data and summary statistics, including contraction time, resting phase, relaxation time, and contraction frequency (*Figure 1A*). To the best of our knowledge, Camystat provides one of the most comprehensive reporting modules currently available in open-access cardiomyocyte analysis software. To achieve these objectives, we designed a custom high-throughput differential analysis algorithm. The differential analysis is an imaging approach that quantifies cardiomyocyte contractile activity by performing bitwise XOR operations between binarized consecutive video frames to detect motion. The number of pixels that change state between frames reflects the intensity of cellular movement over time, allowing visualization and quantification of contraction–relaxation cycles. Moreover, the characteristic shape of the activity curves generated by our algorithm enables automatic identification of contraction and relaxation phases, thereby allowing a high degree of automation in data analysis (*Figure 1B*). To obtain a diverse set of recordings for algorithm design and validation, human iPSCs (UKKi036-A, EBiSC) were differentiated into cardiomyocytes using two established Wnt-modulation protocols (Lian *et al*. and Burridge *et al*.) under feeder-free conditions. Spontaneous contractions appeared between days 7 and 12, and recordings were performed on days 20–24 using Zeiss Axio Observer microscopes (30–100 fps) under controlled incubation conditions.^6,7^ To evaluate the consistency of our approach with alternative algorithms, we identified the leading techniques described in the literature. Among these, we selected Brightness Variation, Optical Flow (Lucas–Kanade and Horn–Schunck variants), and Motion Vector Analysis as the most widely represented methods in published studies. We verified the consistency of the algorithm outputs using Spearman’s rank correlation across 25 high-FPS recordings of cardiomyocytes with varying morphologies, each lasting several tens of seconds. The results, summarized in *Figure 1D*, demonstrated a high degree of consistency between our algorithm and those reported in the literature (with the exception of the Brightness Variation method), as well as accurate representation of the contraction–relaxation cycle waveform characteristics (*ρ* > 0.75; *P* < 0.001). To compare computational efficiency, 100 iterations of each algorithm were performed on three reference recordings under identical hardware conditions. The Brightness Variation method proved to be the fastest (x̄ = 33.3 s), while all other algorithms demonstrated similar performance, ranging from approximately 50 to 59 s per run. Overall, computation times exhibited minimal variance (σ < 0.5 s), confirming stable performance across methods. These results are illustrated in plot 1E, which visualizes the distribution of computation times for each algorithm. In the case of the Horn–Schunck method, no comparative analysis was performed because preliminary tests showed that running this algorithm on a CPU instead of GPU leads to more than an order-of-magnitude increase in computation time, making a direct comparison under such non-optimal conditions unjustified. Contractile activity was automatically analysed with Camystat and compared against manual human measurements as the reference standard. The algorithm correctly identified contractile activity in 23 of 24 recordings, showing high agreement with manual results except in cultures with multiple contraction centres or extremely small active areas. Although parameter adjustment was occasionally required, the algorithm demonstrated high sensitivity and specificity within defined conditions. However, Camystat has several limitations that should be addressed in future releases. The algorithm’s performance remains dependent on parameter adjustment tailored to the characteristics of individual recordings, which limits full automation in settings with high variability of imaging data. The current computational efficiency is stable and comparable to other methods used for contractility assessment. However, the computational profile of differential imaging techniques indicates that further improvements in throughput are feasible. Incorporating GPU-accelerated processing in future versions of Camystat would substantially reduce analysis times and enhance the scalability of the tool. These limitations outline clear directions for continued refinement without compromising the reliability of the present findings.

**Figure 1:**
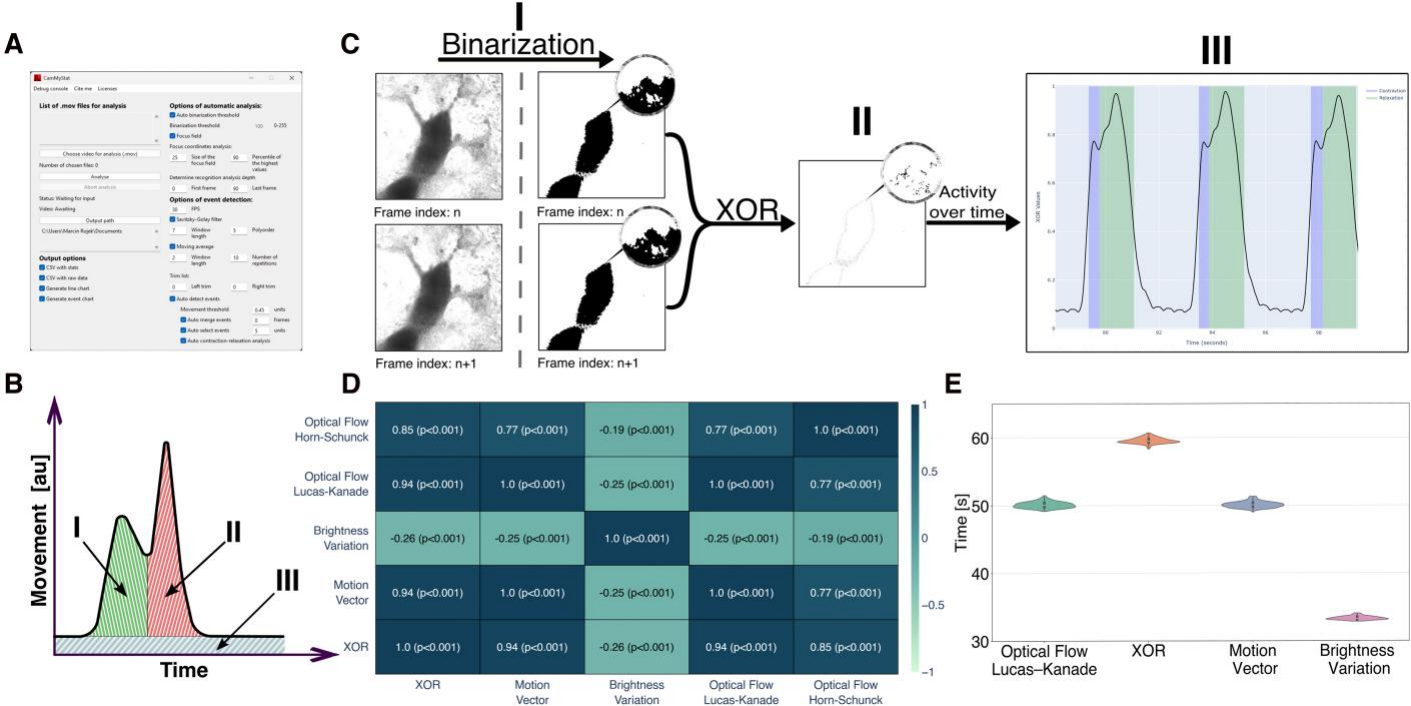
(*A*) Interface of the Camystat software running on Windows. Shown are the available analytical modules and export options, including raw output (CSV), interactive plots, heatmaps, and summary statistical reports. (*B*) Schematic representation of a single contraction–relaxation cycle: (I) area under the curve corresponding to the contraction phase; (ii) area under the curve corresponding to the relaxation phase; (iii) baseline noise floor between contractile events. (*C*) Differential motion-analysis workflow. Consecutive frames (n and n + 1) are first binarized (i), after which inter-frame displacement is quantified using an XOR operator (ii). The final panel presents a representative motion-derived signal generated by Camystat, illustrating temporal changes in pixel-level differences (iii). (*D*) Spearman rank-correlation heatmap comparing image-analysis algorithms. Correlations were computed using 61 388 paired measurements derived from all frames across 25 independent cardiomyocyte recordings (frame-level observations treated as technical replicates nested within recordings). (*E*) Comparison of computational performance across algorithms. Each distribution represents execution times obtained from 100 repeated runs per recording across three independent reference recordings (total *n* = 300 measurements; technical replicates nested within recordings).The brightness variation method exhibited shorter execution times compared to other approaches.

In summary, Camystat provides a standardized, open-source platform for the quantitative assessment of cardiomyocyte contractility, bridging the gap between fully manual and highly specialized proprietary solutions. By integrating automated motion detection, temporal and spatial activity profiling, and reproducible output reporting, the software facilitates robust evaluation of cardiomyocyte function across experimental conditions and laboratories. We anticipate that Camystat will contribute to improving the reproducibility of *in vitro* cardiotoxicity screening and functional characterization of stem cell–derived cardiomyocytes, ultimately supporting translational applications in cardiovascular research.

## Data Availability

The data underlying this article are available in the CAMYSTAT GitHub repository and can be accessed directly via the provided URL.
